# Electrophysiological Evidence for Elimination of the Positive Bias in Elderly Adults with Depressive Symptoms

**DOI:** 10.3389/fpsyt.2018.00062

**Published:** 2018-03-05

**Authors:** Huixia Zhou, Bibing Dai, Sonja Rossi, Juan Li

**Affiliations:** ^1^Center on Ageing Psychology, CAS Key Laboratory of Mental Health, Institute of Psychology, Beijing, China; ^2^Department of Psychology, University of Chinese Academy of Sciences, Beijing, China; ^3^Magnetic Resonance Imaging Research Center, Institute of Psychology, Chinese Academy of Sciences, Beijing, China; ^4^State Key Laboratory of Brain and Cognitive Science, Institute of Biophysics, Chinese Academy of Sciences, Beijing, China; ^5^Institute of Psychology, Tianjin Medical University, Tianjin, China; ^6^Clinic for Hearing-, Speech- and Voice Disorders, Medical University of Innsbruck, Innsbruck, Austria

**Keywords:** elderly with depressive symptoms, cognitive theories of depression, positive bias, event-related brain potentials, late positive potential

## Abstract

**Background:**

Depressed populations demonstrate a greater tendency to have negative interpretations on ambiguous situations. Cognitive theories concerning depression proposed that such a negative bias plays an important role in developing and maintaining depression. There is now fairly consistent evidence arising from different stimuli and assessment methods that depression is featured by such a bias. The current study aimed to explore the neural signatures associated with the interpretation bias in the elderly with depressive symptoms confronted with different facial expressions using event-related brain potentials (ERPs).

**Methods:**

Participants were 14 community-dwelling older adults with depressive symptoms assessed by the Center for Epidemiologic Studies Depression scale scores. We collected event-related potentials of their brain compared to that of 14 healthy aged-matched adults. The late positive potential (LPP) was used to examine cognitive-affective processes associated with judgment of emotional facial expressions between the two groups.

**Results:**

Old adults with depressive symptoms have much smaller amplitude than healthy older adults irrespective of the prime types. When processing the targets, the two groups showed different patterns regarding the LPP. The healthy control group revealed no differences between ambiguous and happy primes, irrespective of whether the targets were sad or happy facial expressions. However, significant differences were found between happy and sad and between ambiguous and sad primes. Such a pattern indicates a positive bias in healthy elderly adults. Regarding the elderly with depressive symptoms, there were no significant differences between ambiguous versus happy, ambiguous versus sad primes, and happy versus sad primes. Concerning reaction times, there was no group difference. Thus, the findings provide some support for cognitive theories of depression.

**Conclusion:**

The current study shows that there is an association between interpretative biases and depressive symptoms in the elderly by using the neuroscientific method of ERPs. The results suggest that ERPs are sensitive to explore the interpretation bias in depressed populations.

## Introduction

The predominant symptoms of depression include negative beliefs about the world, the self, the future, as well as periodical and unmanageable negative thoughts which frequently linger around the self. Cognitive theories of depression proposed that depressed populations show a greater tendency to have negative interpretations on ambiguous stimuli, situations, and events (1). According to cognitive theories of depression, such a negative interpretation bias is assumed to play a central role for both development and maintenance of depression (1, 2). There is now fairly consistent scientific evidence concerning the interpretative biases in depression. Previous studies have employed various methods to explore this issue, the most prevalent being self-reports of subjects’ interpretations of scenarios and stories with ambiguity (3–5). Such methods substantially contributed to the establishment of cognitive theories of depression. Even so, as Lawson and MacLeod (6) put forward, self-reporting methodologies are vulnerable to effects caused by response bias. For instance, depressed individuals probably process the negative and neutral interpretations of ambiguous information in a similar way to non-depressed ones but show a greater inclination to give the more negative interpretations.

In order to circumvent this problem, many studies switched to the usage of performance-based measures, such as priming tasks, but failed to discover interpretation biases in populations with depression (6–8). Some researchers proposed that the failure in finding interpretive biases through priming methods possibly owning to the application of reaction times (RTs) measures to evaluate the cognitive processing of depressed individuals (9). Distinctively, severity of depression is correlated with both the retardation and increased variability of the response latencies to carry out voluntary reactions. Thus, response latencies are not sensitive enough to detect interpretive biases in depressed individuals. To resolve the problems related to response latencies in priming studies, Lawson et al. (9) used physiological indices, like the magnitude of the human startle reflex. They found that depressed people showed a similar eye blink reaction elicited by negative and ambiguous words, but the blink reflexes magnitudes to ambiguous words was much smaller than those to negative words in healthy controls. Previous studies have found that magnitude of the startle reflex was increased after the presentation of negative stimuli (10, 11). Therefore, such findings are consistent with the hypothesis of a negative interpretation bias in depressed individuals.

The heterogeneity of results probably, at least was partially caused by methodological difficulties in evaluating the biased information processing in depression. Given this issue, it is of great necessity to adopt an alternative method, which should not use measurement reflecting speed to execute voluntary responses, and also must avoid the potential impact from response bias effects. As a neurophysiological measure assessing online brain processing mechanisms, event-related brain potentials (ERPs) bear the potential to overcome the limitations mentioned above, providing a unique chance to explore how depressed populations preliminarily process incoming information. Furthermore, evidence from ERPs might be of essential importance to cognitive processing theories of depression (12–14). In addition, featured by high temporal resolution, ERPs can directly measure the neural activity occurred just before the elicitation of behavioral response. A positive ERP component beginning about 300 ms after the stimulus onset has been consistently associated with arousal and emotion (15). Put forward by Kissler et al. (16), this component has been named as the late positive potential (LPP). Studies have demonstrated that the LPP tend to be augmented for emotional stimuli (17, 18). Furthermore, this potential was also found to be correlated with subjective ratings of emotion intensity (19). Amusingly, some researchers have also found that LPP can differentiate negative stimuli from positive ones (20).

The majority of previous studies try to explore the interpretation bias in individuals with depression by using words, sentences, scenarios, and events with ambiguity as experimental stimuli. Such stimuli are loaded with low levels of emotion, thus have poor sensitivity and ecological validity. Recently, considerable empirical researches have used faces with emotional expressions as experimental materials (21, 22). Emotional faces have the following advantages when compared with those used in the majority of previous studies. First, emotional faces are less influenced by different cultural background and people come from different ethnic group incline to interpret basic facial expressions in a similar way (23). Second, facial expressions, as one of the most important message sources in the ongoing stream of various social cues during social interactions, can pass on a wide-spreading of information among social partners (24), about 60% of information (25). Third, interpersonal theories of depression proposed that depressed individuals elicit rejection from others in their social interaction, which in turn exacerbates their future risk of suffering from depression (26). Fourth, faces with emotional expressions are closely allied to the estimations of social approval or disapproval (27) hence it is essential to precisely interpret and react to them for effective social functioning (28). Last but not least, as individuals usually attempt to control their emotional expression in daily interaction, it is quite common to see mild or ambiguous facial expressions. Therefore, it is very difficult to aware and interprets these ambiguous social signals. As a result, it would be more sensitive to use ambiguous facial expressions to explore the interpretive bias in depressed populations. By using facial expressions, some previous studies have detected the interpretation bias in depressed individuals (21, 27, 29).

Individuals with depressive symptoms reside in a stage during which psychometrically identified depression is above the average. However, they do not fulfill the diagnostic criteria of clinical depression overall. Thus, they lie in the middle of a continuum between normal mood and clinical depression. As for preclinical depression, adults with depressive symptoms probably then develop into clinical depression (30). Investigating populations with depressive symptoms allows studying both susceptibility and compensation mechanisms of depression (31). As a consequence, exploration of such mechanisms would enhance our comprehension of the underlying processes bring about to clinical relevant depression. Detection of ERPs biomarkers of individuals with depressive symptoms will have important illuminations for early diagnosis of risk populations thus possibly preventing depression onset.

Studies concerning adults and adolescents have revealed that depression and negative interpretation bias is closely correlated, however, its emergence in the elderly still remained unknown due to the discrepancy between young and older adults in depression manifestation and emotion processing (32, 33). First, the elderly with depression showed more sleep disorder and loss of appetite when compared with depressed adults and adolescents [National Institutes of Health (34)]. Second, proposed by the socioemotional selectivity theory, healthy old adults would show a positivity bias in emotion processing compared with the younger ones (33, 35).

In summary, the present study aimed to assess the interpretation bias in the elderly with depressive symptoms when making judgments of facial expressions *via* a priming paradigm. For this purpose, ERPs will be used to evaluate the biased information processing in the elderly. According to the socioemotional selectivity theory proposed by Carstensen (33, 35), we hypothesized that older adult without depressive symptoms would exhibit a positive bias in processing emotional faces. Based on cognitive theories of depression, we hypothesized that the elderly with depressive symptoms would show a negative interpretation bias compared with healthy controls. Measured by RTs, previous studies failed to find interpretation bias in depressed individuals. We hypothesized that the ERP component LPP would reflect an interpretation bias in the elderly with depressive symptoms.

## Method

### Participants

All subjects in the current study were chose from the participants’ database of our previous study (36). There are 61 elderly with depressive symptoms and 245 healthy normal controls (NCs) in the database. First, 14 subjects were randomly selected from those 61 elderly with depressive symptoms. A control group was then randomly selected from the 245 normal old adults. The two groups were matched in their demographic variables.

The average age of the healthy NC group was 65.64 years. There are five males and nine females in this group. The mean age of the older adults with depressive symptoms (ADs) was 66.36 years. There are also five males and nine females in this group. The two groups have no difference in their mean age (*p* = 0.71) or education (*p* = 0.78). The Center for Epidemiologic Studies Depression Scale (CES-D) (37) was applied to obtain each participant’s depression scores. There are 20 items in the CES-D, and participants are asked to self-rate the presence of their depressive symptoms during the past week on a 4-point Likert-type scale. The total score of the CES-D is 60. Participants with a score of 16 or greater were considered to have depressive symptoms. Studies have shown that the CES-D possess well-established psychometric attributes with older adults (38). The mean CES-D score for the ADs group was 20.21 (SD = 5.65) and that for the NC group was 3.5 (SD = 3.3). The ADs participants had a Mini-Mental State Examination (MMSE) cutoff of ≥24, and they did not meet the DSM-IV clinical criteria for major depressive disorder (MDD). The NC participants had a CES-D cutoff of ≤5 and a MMSE score ≥24.

Written informed consent was obtained from all participants to study participation. They received a compensation for their participation. They all declared that they had no neurological and psychiatric diseases. All participants were right-handed and had normal or corrected-to-normal vision. The clinical and demographic characteristics of these participants are shown in Table 1.

**Table 1:** The clinical and demographic characteristics of the participants.

Characteristics	NC	ADs	*p*-Value
Age (years)	65.64 ± 3.93	66.36 ± 5.20	0.71
Education (years)	12.71 ± 3.29	12.00 ± 3.26	0.78
MMSE	28.64 ± 1.65	27.57 ± 1.91	0.12
CES-D	3.5 ± 3.30	20.21 ± 5.65	<0.01

### Stimuli

Digitalized photographs of affective expressions with both male and female model identities were taken from the Chinese Facial Affective Picture System. The photographs included 870 emotional faces (39). By using the morphing software (Morpheus v. 1.95), affective expressions of each model identity were then blended into each other to create a 50% happy and 50% sad intensity level of ambiguity. Emotional faces of five male and five females with happy and sad expressions, as well as five 50% happy–50% sad ambiguous expressions were applied as primes in the current study. Therefore, there were three kinds of primes, namely happy, sad, and ambiguous facial expressions. Moreover, another series of emotional faces of five male and female with happy and sad expressions were selected from the same Chinese Facial Affective Picture System and used as targets, which resulted in two kinds of targets, happy and sad faces. Hence, the stimulus material for the experiment consisted of 300 distinct images, with 50 happy–happy pairs, 50 happy–sad pairs, 50 sad–happy pairs, 50 sad–sad pairs, 50 ambiguous–happy pairs, and 50 ambiguous–sad pairs. These stimuli were split into five blocks, with 60 trails in each block. A separate set of stimuli was created in the same manner for the practice trials. All pictures were situated within an area of 184 × 198 pixels. Picture size was 7.9 × 7.3 cm^2^, and they were shown in the center of the computer screen.

### Procedure

Participants with informed consent were given instructions on how to prepare for the ERP recording. In the formal experiment, participants were stayed in a room with dim lighting, sound insulation and electric shield. In each trial, a fixation cross of 400 ms was presented first. Then, a prime face appeared for 200 ms, which was followed by a 100 ms interval. Finally, the target face was presented for 2,000 ms. Following the target face, a blank screen was presented for 1,500 ms, during which the participants were allowed to blink. Participants were required to classify the target face as either “happy” or “sad” as fast and accurately as possible by pushing one of the two horizontally arranged buttons with their index fingers. The face-to-hand assignment was counterbalanced across participants.

### Electrophysiological Recordings

The electroencephalogram (EEG) was recorded with a 64 Ag/AgCl cap placed in referring to the extended 10–20 positioning system (http://www.neuroscan.com/). EEG signal was recorded with 500 Hz sampling rate and referenced to the right mastoid (M2) online. Impedances were kept below 5 kΩ. During recording, a 30 Hz low-pass filter was used. The eye blinks of participants were corrected mathematically. The remaining artifacts were rejected manually. For the LPP component, segments of 1,000 ms beginning 200 ms pretarget and ending 1,000 ms after target onsets were obtained. A 200 ms pretarget baseline was used to average the segments in order to acquire ERPs. Signals exceeding ±80 µV in any given epoch were discarded automatically.

### Behavioral Data Analysis

The judgment RTs were analyzed, while accuracy rates were ignored because of the high accuracy (>95%). The analysis of variance (ANOVA*)* performed on RTs revealed a significant main effect of prime, *F* (1, 26) = 4.48, *p* < 0.05, η^2^ = 0.26, with judgment latencies of ambiguous primes (617.15 ± 17.91 ms) being faster than that of both happy (633.97 ± 15.98 ms) and sad primes (639.77 ± 18.83 ms), but the happy and sad primes did not differ from each other. Furthermore, no other main effect or interactions approached significance.

### ERP Data Analysis

The mean amplitudes in the time range 350–650 ms (LPP) were then exported. According to previous studies and on account of visual inspection of the present data, we focused the analysis of the LPP in this time range over three regions of interest, i.e., frontocentral: FC1, FC3, FCz, FC2, and FC4; centroparietal: CP1, CP3, CPz, CP2, and CP4; parietooccipital: PO3, PO5, POz, PO4, and PO6.

We calculated an ANOVA with the factors prime (happy, sad, and ambiguous), target (happy, sad), and topographical region (frontocentral, centroparietal, and parietooccipital) as a within-subject factor, and group (NC, ADs) as a between-subject factor.

**Figure 1:**
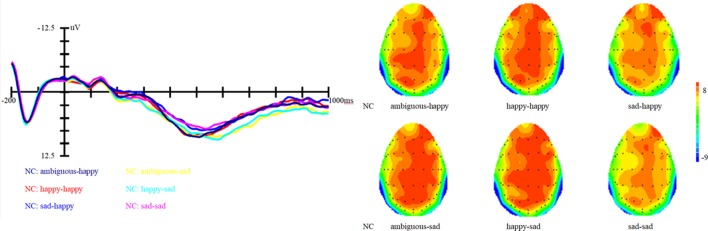
Grand average event-related brain potentials at the electrode CPz of the analyzed topography to ambiguous, happy, and sad facial expressions for both the happy and sad target face for healthy old adults.

**Figure 2:**
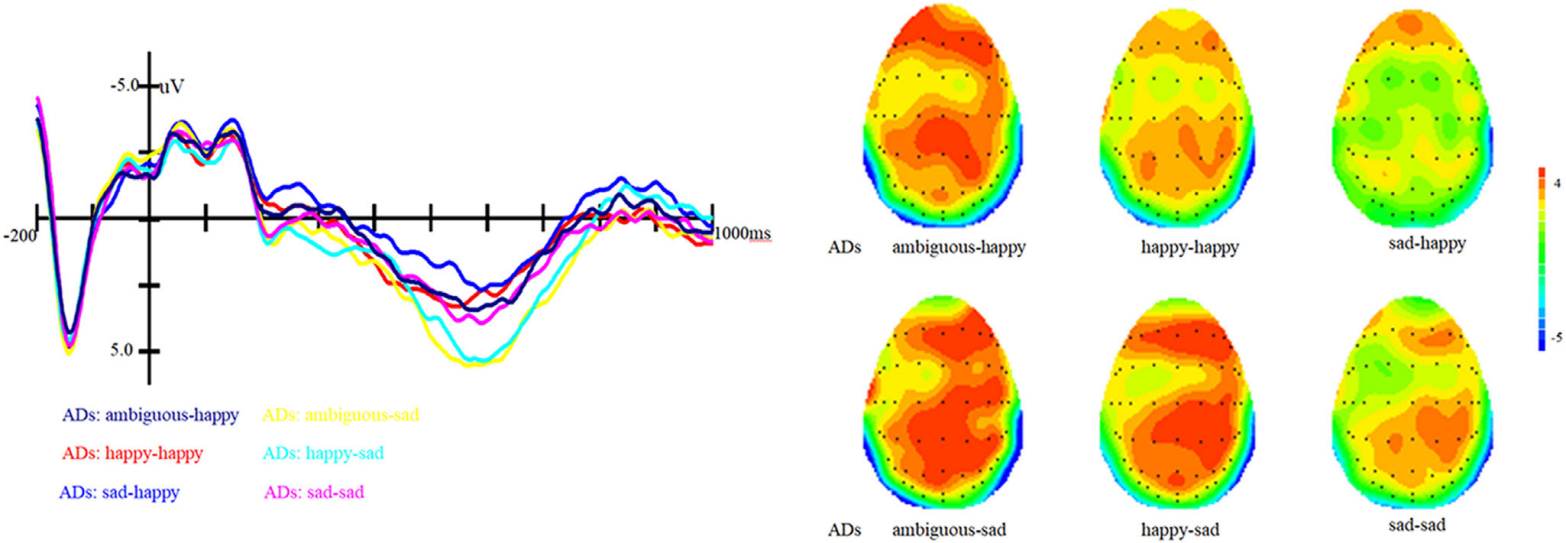
Grand average event-related brain potentials at the electrode CPz of the analyzed topography to ambiguous, happy, and sad facial expressions for both the happy and sad target face for old adults with depressive symptoms.

Results showed that there is a significant main effect of group, *F* (1, 27) = 16.8, *p* < 0.001, η^2^ = 0.17; target, *F* (1, 82) = 13.66, *p* < 0.001, η^2^ = 0.14 and topographical region, *F* (1, 81) = 39.35, *p* < 0.001, η^2^ = 0.49. Moreover, the interaction between prime and target also approached significance, *F* (1, 81) = 51.59, *p* < 0.001, η^2^ = 0.56. Simple effect analysis showed that no matter the target was happy or sad faces, there were no significant differences between happy and ambiguous primes, but significant differences were present between happy versus sad and ambiguous versus sad primes, with amplitudes of happy primes larger than that of sad primes (*p* < 0.05), and also larger amplitudes for ambiguous compared to sad primes (*p* < 0.05). ERP waveforms and topographic maps (time range: 350–650 ms) of both sad targets and happy targets for two groups of participants are shown in Figures 1 and 2.

**Figure 3:**
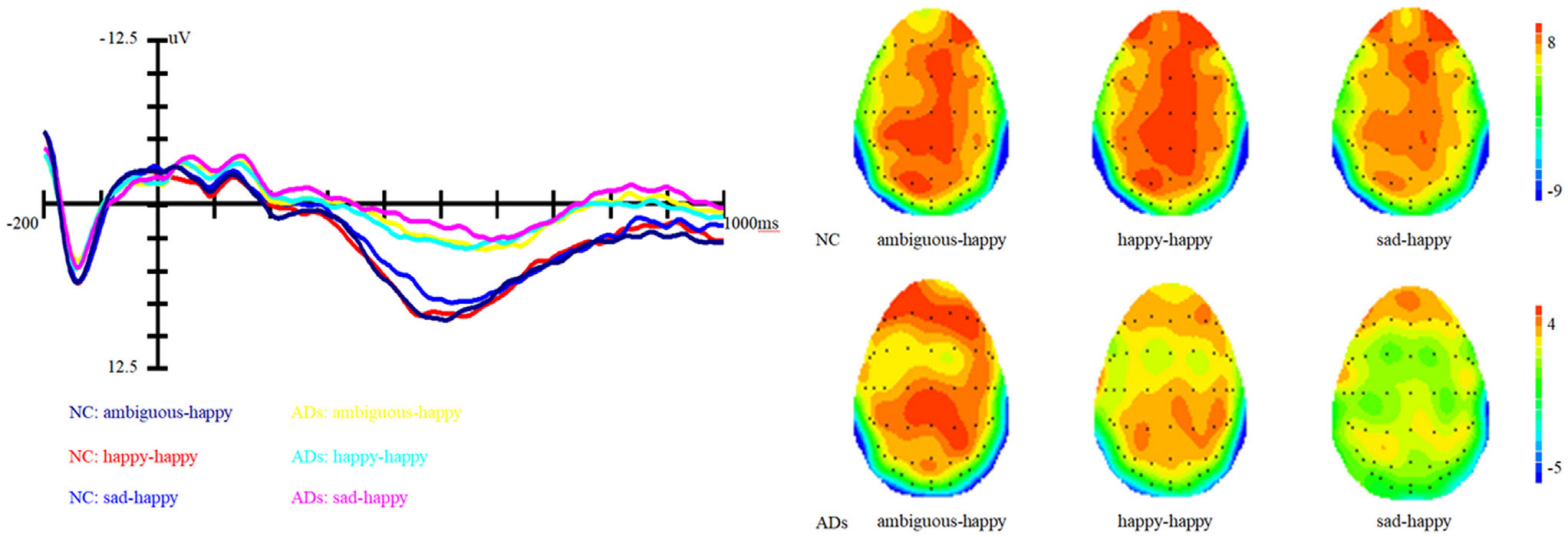
Grand average event-related brain potentials at the electrode CPz of the analyzed topography to ambiguous, happy, and sad facial expressions when the target was a happy face for both the healthy old adults and old adults with depressive symptoms.

**Figure 4:**
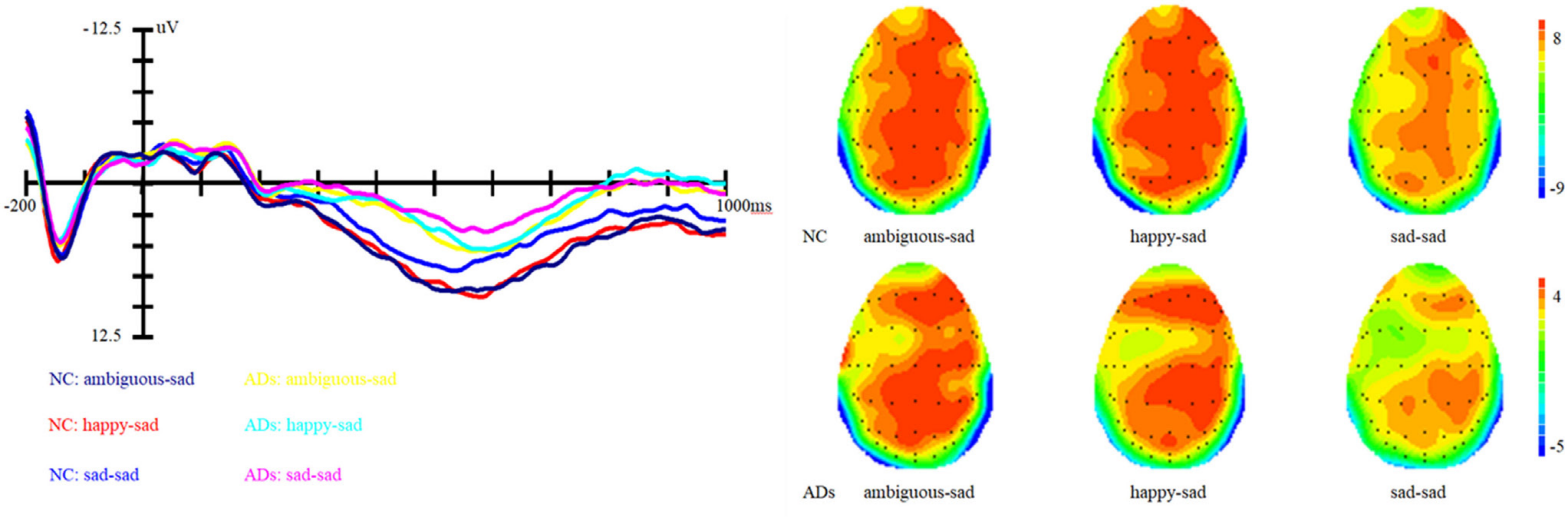
Grand average event-related brain potentials at the electrode CPz of the analyzed topography to ambiguous, happy, and sad facial expressions when the target was a sad face for both the healthy old adults and old adults with depressive symptoms.

In addition, the interaction between prime and group is marginally significant, *F* (1, 81) = 2.62, *p* = 0.07, η^2^ = 0.06. Simple effect analysis showed that ADs have much smaller amplitude than healthy older adults, irrespective of the prime types (all three *p*s < 0.01). For healthy older adults, happy primes did not differ from ambiguous primes (*p* > 0.05), but significant differences were present between happy versus sad and ambiguous versus sad primes, with amplitudes of happy primes larger than that of sad primes (*p* < 0.05), and also larger amplitudes for ambiguous compared to sad primes (*p* < 0.05). For ADs, there were no significant differences between happy versus sad, ambiguous versus sad, and ambiguous versus happy primes (all three *p*s > 0.05). Grand-average ERP waveforms and topographic maps (time range: 350–650 ms) for both groups of participants are shown in Figures 3 and 4.

## Discussion

By using ERPs, the current study aimed to assess the interpretive bias in the elderly with depressive symptoms when making judgments of facial expressions. We hypothesized that the elderly with depressive symptoms would show a negative interpretation bias compared with healthy controls. Results of the current study demonstrated that elderly adults with depressive symptoms have much smaller amplitude than healthy older adults irrespective of the prime types. When processing the targets, the two groups showed different ERP patterns concerning the LPP. The healthy control group revealed no differences between ambiguous and happy primes, irrespective of whether the targets were sad or happy facial expressions. However, significant differences were found between happy and sad and between ambiguous and sad primes. Regarding the elderly with depressive symptoms, there were no significant differences between ambiguous versus happy, ambiguous versus sad primes, and happy versus sad primes. Again, these modulations occurred in a similar strength irrespective of whether a sad or happy target was presented. Concerning reactions times, there was no difference between the two groups.

The LPP reflects a late and controlled attentional evaluation of emotion. Results of the present study with respect to the LPP showed differences between healthy old adults and elderly with depressive symptoms. The LPP findings reveal that normal old adults were more possibly to identify ambiguous facial expressions as happy ones. Such a pattern indicated that there was a positive bias in the normal old adults. This result provided evidence for Carstensen’s socioemotional selectivity theory (33, 35), which proposed that normal older adults showed positive preferences for positive over negative emotional information (33, 40). Nevertheless, these characteristics might not apply to the elderly with depressive symptoms. Perhaps, elderly adults with depressive symptoms cannot make a distinction between ambiguous, happy and sad faces, which might suggest an elimination of the positive bias.

Compared to the positive bias observed in healthy controls, the elimination of such a bias in elderly adults with depressive symptoms could also be considered as a kind of negative trend. Therefore, the present study further confirms that there is a depression-linked interpretive bias in depressed individuals and provides further evidence for the cognitive theories of depression (1, 2). However, result of the current study is different from that of Lawson et al. (9). They used the human eye blink reflex to explore the negative interpretation bias in depressed college students and found that depression is associated with a negative interpretation bias. The current study, similar to Lawson et al. (9), used a method which does not depend on subjects’ self-reports and can also evade the troubles related to response latency measurement of cognitive processing in individuals with depression, only found an elimination of the positive bias but not a negative bias. It is essential to notice that the difference between our findings and that of Lawson et al. (9) might have been potentially caused by factors such as differences in demographic characteristics of the participants and the kind of depression measures. In Lawson et al. (9), undergraduate students were selected as participants. While in our study, old adults were recruited as participants. Actually, Wood and Kisley (41) had found that old adults and young adults are quite different in processing emotional information. Such differences in participant samples may contribute to the inconsistent findings. Concerning the use of different measurement tools to assess depression, Lawson et al. (9) selected participants with scores on the Beck Depression Inventory. In contrast, we used CES-D to define the depressive symptoms in the elderly. Such differences in measuring depression may also contribute to the inconsistent results. The inconsistency between our study and Lawson et al. (9) also implicated the pressing need for more studies on interpretation bias associated with depression. The current study just catch a glimpse of the neural mechanisms underlying depression, thus future research should explore other related aspects so as to obtain a more integrated understanding of the potential neural underpinnings of the interpretation bias in depression.

The current study demonstrated that the LPP amplitude of elderly adults with depressive symptoms is significantly smaller than that of the healthy older adults. Although the interaction between group and target is marginally significant, simple effect analysis showed that the two groups of participants showed different LPP patterns irrespective of whether a sad or happy target was presented. Concerning such findings, the possible reason is that the sample size of the current study is relatively modest. However, one thing for sure is that depressive symptoms really have some influences on the LPP amplitude of old adults. Therefore, a large sample would be employed in the future to validate the findings of the current study.

## Conclusion

In summary, the present study found that there exists a positive bias in the healthy elderly. However, depressive symptoms in the elderly not only caused a reduction of LPP amplitude but also an elimination of the positive biases. Such findings highlighted a relation between interpretative biases and depressive symptoms in the elderly by using the neuroscientific method of event-related potentials. The results suggest that ERPs are sensitive to explore the interpretation bias in depressed populations, which enhanced our understanding of the underlying processes bring about to clinical relevant depression and also have important illuminations for early diagnosis of at risk populations. In addition, the present study expanded research concerning interpretation bias to the elderly.

## Ethics Approval and Consent to Participate

The current study was approved by the Ethics Committee of the Institute of Psychology, Chinese Academy of Sciences. Written informed consent of participants was obtained for participation after a detailed explanation of the study procedure.

## Availability of Data and Materials

The data and materials supporting this study cannot be made publicly available because it will be in conflict with patients’ privacy. All interested parties can request the data from lijuan@psych.ac.cn.

## Author Contributions

JL, HZ, and BD initiated the design for this article together. HZ undertook the statistical analysis and wrote the manuscript. BD participated in the data collection and also made some critical comments on the manuscript. SR made critical comments and revision on the manuscript. As the principal investigator of this project, JL supervised the statistical analysis and the manuscript writing and revision. All authors read and have approved the final manuscript.

## Conflict of Interest Statement

The authors declare that the research was conducted in the absence of any commercial or financial relationships that could be construed as a potential conflict of interest.

## Abbreviations

ERPs, event-related brain potentials; LPP, late positive potential; NC, normal control; ADs, older adults with depressive symptoms; CES-D, Center for Epidemiologic Studies Depression scale; MMSE, Mini-Mental State Examination; MDD, major depressive disorders; EEG, electroencephalogram; ANOVA, analysis of variance; RTs, reaction times.
